# Experiences of post-surgery exercise among middle-aged adults with early lung cancer

**DOI:** 10.1515/med-2026-1467

**Published:** 2026-07-27

**Authors:** Ka Ryeong Bae, Garam Bang, Genehee Lee, Juhee Cho, Sunga Kong

**Affiliations:** College of Nursing, Eulji University, Seongnam, South Korea; R&D Supporting Team, Samsung Medical Center, Seoul, South Korea; Patient-Centered Outcomes & Value Enhancement Institute, Samsung Medical Center, Seoul, South Korea; Department of Clinical Research Design and Evaluation, SAIHST, Sungkyunkwan University, Seoul, South Korea; School of Medicine, Sungkyunkwan University, Seoul, South Korea; Future Medical Research Institute, Samsung Medical Center, Seoul, South Korea

**Keywords:** cancer survivors, physical activity, recovery of function, health behavior, qualitative research

## Abstract

**Objectives:**

This study aimed to explore post-surgery exercise experiences of middle-aged early lung cancer survivors. This qualitative study used phenomenological methods reflecting Merleau-Ponty’s perspectives of the body.

**Methods:**

The participants were 22 adults (aged 40–64) with stage I non-small cell lung cancer, who underwent surgery at a university hospital in Seoul, South Korea, between May and October 2017. Data were collected through individual in-depth interviews. Narrative meaning was constructed and basic structure of the research topic was confirmed.

**Results:**

Three themes and eight subthemes captured the post-surgery exercise experiences of middle-aged early lung cancer survivors: “quickly regaining one’s daily life at a time of turmoil,” “efforts to exercise in a healthy manner,” and “conflict between the need to exercise and practical difficulties.” Participants quickly returned to their daily lives post-surgery and tried to participate in smart exercise regimes to prevent recurrence. However, they experienced obstacles such as psychological pressure, lack of time, and difficulty continuing exercise, due to lack of specific exercise information and guidelines for patients with lung cancer.

**Conclusions:**

Customized exercise interventions and education programs considering patients’ physical condition and lifestyle environment need to be developed in both clinical and community settings.

## Introduction

Globally, with 2.22 million new cases and 1.55 million deaths annually, lung cancer is a critical health burden [1]. Approximately 85 % of lung cancer cases are non-small cell lung cancer (NSCLC). Because the average age at diagnosis is 70 years, lung cancer is traditionally viewed as an old person’s disease; however, the average age has been gradually decreasing [2]. While the proportion of lung cancer patients younger than 40 years remains relatively low (less than 10 %), the incidence of lung cancer is gradually increasing in younger and middle-aged people, especially in the 40–65 age group. This growing incidence among younger patients underscores the need for research on treatment, prognoses, and outcomes in this demographic [3]. Middle-aged patients who have received an early lung cancer diagnosis are likely to survive and live long. Interventions are often focused on health management programs for patients who need to continue economic pursuits; thus, there is an urgent need to develop tailored programs for these patients. [4].

Currently, surgery is the best treatment option for patients with early NSCLC [5]. After radical resection, 5-year survival for patients with stage I NSCLC is known to increase by up to 70 % [6]. Despite the increased survival rates, the quality of life (QoL) of patients with NSCLC after lung resection is much lower than that of other patients with cancer. Owing to the sudden decline in lung function, patients experience dyspnea, pain, and physical dysfunction [7], [8], [9], and report poor long-term QoL. However, in a prior study by the authors, cardiopulmonary function can be recovered within six months of lobectomy, and both pre- and postoperative physical activity levels are important for this recovery. In particular, moderate-to-vigorous physical activity in the immediate postoperative phase is essential, highlighting the need for exercise [10].

Studies have demonstrated that pre-lobectomy cardiopulmonary function is a preventive factor against complications [11], and that exercise programs shortly after lobectomy can help with the rapid recovery of physical function and improved QoL [12], [13], [14]. With an increase in the number of patients with early lung cancer owing to early testing and diagnosis, there is increasing interest in pre- and postoperative management programs. However, to establish customized post-surgery exercise programs for patients with lung cancer, guidelines and strategies are needed that consider reduced pulmonary and physical function, including symptoms caused by surgery [15]. Although recent studies have examined the stability and efficacy of exercise programs for patients with lung cancer [16], most of these were quantitative studies assessing the effects of such programs. To improve compliance with exercise programs and program effectiveness, it is crucial to consider the meaning of exercise to patients as well as their needs and knowledge level. A key advantage of qualitative research is that it helps researchers listen to and reflect on patients’ thoughts, which is a prerequisite for developing programs applicable to specific patient groups.

Existing qualitative studies on exercise in lung cancer have primarily focused on patients with metastatic or advanced disease [17], 18]. One longitudinal feasibility study examined participation in an exercise intervention initiated immediately after surgery [19], while others have explored patients’ and health professionals’ views on exercise interventions for surgically treated patients [20] or patients’ experiences of participating in structured home-based exercise programs [21]. However, these studies largely addressed intervention feasibility or acceptability within specific program contexts. Few studies have specifically explored how middle-aged adults (aged 40–64) with early-stage lung cancer experience and perceive self-directed exercise after curative surgery, including their practical barriers, unmet information needs, and preferences for health management in everyday life.

Therefore, this study sought to understand the experience of various post-surgery exercises among middle-aged patients with early-stage lung cancer. The study aims to provide a basis for the development of multifaceted programs to promote physical activity and improve patients’ pre- and postoperative QoL. The specific research question was, “What are the experiences of middle-aged patients with early lung cancer regarding post-surgery exercise, and what are their unmet needs?”

## Methods

This study adopted a phenomenological approach reflecting Merleau-Ponty et al.’s perspectives of the body [22] to explore and elucidate the experiences of postoperative exercise among middle-aged patients with early lung cancer. Merleau-Ponty’s phenomenology considers the body as “being in the world” (être au monde), and consciousness, which cannot be separated from the body, is also “being in the world.” By always being in the world, the body forms a relationship with the world based on the exchange of directional meaning, and people go out into the world to form new relationships involving such exchanges of meaning. The concept of the world, perceived through the body, is considered a phenomenological space. Merleau-Ponty et al. argued that empiricism and intellectualism, according to which a perceived object can be observed objectively to completely elucidate its nature, have overlooked the constraints imposed on humans by their bodies. In this study, this perspective informed our attention to how participants experienced bodily changes after surgery, how these changes affected their everyday lives, and how exercise was understood as a way of regaining bodily familiarity and reconnecting with daily life.

### Participants and ethical considerations

The participants were patients belonging to the Coordinate Approach to Cancer Patients’ Health for Lung Cancer (CATCH-LUNG) cohort, a prospective cohort of preoperative patients with lung cancer at a single university hospital in Seoul between March 2016 and October 2018. The inclusion criteria were (1) age 40–64 years; (2) histological diagnosis of NSCLC IA or IB; (3) lung resection within the last year; (4) no prior chemotherapy or radiotherapy; and (5) comprehension of the study objectives with voluntary consent to participate. The exclusion criteria were (1) prior pneumonectomy; (2) limited cognitive or communication abilities; and (3) current psychiatric treatment. There were 22 participants, of whom 13 were women (59 %). The mean age was 56.14 years (range: 41–64 years). Fifteen participants were diagnosed with NSCLC IA (68 %), while the rest were diagnosed with NSCLC IB (32 %). When the interviews were conducted, the mean time since surgery was 5.18 months (range: 1–12 months). The most common surgical method was lobectomy (16 persons, 73 %; Table 1).

**Table 1:** Participant characteristics (n=22).

Characteristic	n (%)
Age, median (range), years	56.14 (41–64)
Female	13 (59.1)
Cancer stage	
IA	15 (68.2)
IB	7 (31.8)
Postoperative time, median(range), months	5.18 (1–12)
Type of operation	
Lobectomy	16 (72.7)
Wedge resection	3 (13.6)
Other	3 (13.6)

This study was conducted in accordance with the Declaration of Helsinki (as revised in 2013) and was approved by the Institutional Review Board (IRB) of the authors’ affiliated institution (IRB No. 2015-11-025). Written informed consent was obtained from all participants prior to participation. Participants were fully informed of the study objectives, potential risks and benefits, confidentiality protections, and their right to withdraw at any time without consequence. All data were coded, stored securely under password protection, and maintained to ensure participant anonymity.

### Data collection

The interviews were conducted over six months from May to October 2017. Patients were recruited until data saturation. To properly understand patients’ experiences of exercise, the interviews were conducted by S. Kong, a trained expert in sports science, and G. Lee, an advanced practice nurse for lung cancer. Face-to-face individual interviews were conducted to explore patients’ experiences of exercise and their perceptions of the benefits of and barriers to exercise. The interviews were conducted using a semi-structured questionnaire designed by the researchers. The interviews lasted 30 min on average and were conducted in a quiet location in the hospital where patients’ privacy could be protected and the interview content could be kept confidential. The interviews were recorded and transcribed. Disease-related medical data were also collected through electronic medical records.

### Data analysis

The researchers listened to the recordings several times and transcribed the participants’ statements verbatim. The transcribed data were read repeatedly to ascertain the overall essence and meaning of the content. The transcribed content was reviewed to extract meaningful passages or phrases, and these were developed into general, abstract statements to construct meaning. The meanings thus derived were combined to form subthemes and themes and converted to clear statements that represented the fundamental structure of the research topic. Although the coding process was primarily inductive and grounded in participants’ descriptions, Merleau-Ponty’s phenomenology informed the interpretation of the findings by sensitizing the researchers to participants’ embodied experiences, including disruptions in their taken-for-granted bodily routines, efforts to regain everyday functioning, and tensions between bodily vulnerability and the desire to return to ordinary life through exercise. Throughout the research process, the researchers used bracketing to minimize preconceptions and bias and aimed to prevent data distortion by focusing on participants’ experiences and examining the data as they were.

### Ensuring rigor

We followed Lincoln and Guba’s principles of credibility, transferability, dependability, and confirmability [23]. To interpret participants’ original unaltered narratives, the interviewers summarized and explained the interview contents immediately after the interviews and verified with the participants whether the themes matched their statements. Applicability was confirmed by having a thoracic surgeon and an advanced practice nurse for lung tumors verify that the results could potentially have been derived from real middle-aged patients with early lung cancer. Finally, the authors maintained consistency and neutrality by holding regular meetings to review audit trails, re-confirm the analytical process, and perform member checks on theory derivation. The researchers attended classes on qualitative research methodologies at graduate school and continually participated in relevant conferences and seminars to improve their understanding of qualitative research. Moreover, the researchers published several qualitative research papers and have experience developing programs to alleviate physical and mental symptoms and improve QoL among patients with cancer.

### Research ethics

This study was conducted in accordance with the Declaration of Helsinki (as revised in 2013) and was approved by the Institutional Review Board (IRB) of the authors’ affiliated institution (IRB No. 2015-11-025).

### Informed consent

Written informed consent was obtained from all participants prior to participation. Participants were fully informed of the study objectives, potential risks and benefits, confidentiality protections, and their right to withdraw at any time without consequence.

## Results

Based on the main statements extracted from raw data, three themes and eight subthemes were derived (Table 2).

**Table 2:** Post-surgery exercise experiences of middle-aged patients with early lung cancer.

Themes	Subthemes
Quickly regaining one’s daily life at a time of turmoil	Shaken by the unexpected diagnosis
	Quick recovery and return to daily life
Efforts to exercise in a healthy manner	Exercising by considering one’s environment and health
	Smart health management and searching for information
Conflict between the need to exercise and practical difficulties	Lack of information and guidelines for exercise
	Difficulty exercising due to a busy life
	Psychological pressure due to the fear of recurrence
	Mistaken perceptions about exercise

### Theme 1: quickly regaining one’s daily life at a time of turmoil

The participants experienced immense psychological trauma from their cancer diagnosis and found it difficult to lead their daily lives until surgery, due to the fear that they might not recover or might even die. However, the patients quickly recovered after surgery, with almost the same physical ability as before, and did not experience major difficulties returning to daily life.

#### Subtheme 1: shaken by the unexpected diagnosis

The participants experienced extreme confusion and anxiety due to the unexpected diagnosis of lung cancer. Even though they were diagnosed early, the psychological impact of the word “cancer” disrupted their identity and order in life. The participants felt as though their future had become uncontrollable and complained of psychological distress due to fear.

The sky was dark, and I couldn’t say anything… I felt dismal. (Participant 1)

Oh, I won’t be able to live the way I used to. I guess my life’s over now. I felt like I wouldn’t be able to live like this. Really. (Participant 2)

#### Subtheme 2: quick recovery and return to daily life

After surgery, the participants experienced rapid physical recovery and returned to daily life more easily than expected. Although post-surgery discomfort persisted, most participants expressed that they attained preoperative levels of physical ability in a short time.

It’s become much better now. Before, I felt like my stomach would burst, and I had to hold tight here every time I coughed. I don’t have that anymore. I can just cough when I need to and I’ve gotten a lot better. (Omitted) I think I’ve gotten better faster than I expected. (Participant 18)

There’s a fitness room at work. (Omitted). Because we check our body composition, I know that I recovered my body fat and my overall condition to how it was before. I can see a graph of changes since before surgery. (Participant 3)

The participants reported no particular difficulties continuing their daily lives. Therefore, after surgery, they were able to rapidly resume their prior activities and easily return to daily life.

There wasn’t anything specific [that was difficult after surgery]. I felt light even when I went home and I didn’t have any problems. (Participant 10)

I participate in office life. Within just one week of the surgery, I picked up our child. I actually think I’m thriving more than before. (Participant 13)

### Theme 2: efforts to exercise in a healthy manner

Most participants treated exercise as their top priority post-surgery. They made efforts not only for the sake of exercise but also to prevent cancer recurrence and live healthier. They reacted sensitively to the environment where they exercised, and being from a generation comfortable with using the internet, smartphones, and wearable devices, they often used various exercise techniques employing these items.

#### Subtheme 1: exercising by considering one’s environment and health

Many participants sought clean air outdoors when exercising, rather than the gym or the track. Most commonly, the participants went hiking in the mountains. Some left the city and settled in the countryside so that they could walk or climb the mountains several times a day.

I just thought the air is good in nature, so it must be good for my body. I think the low mountains in the neighborhood are just right. First, even if it’s a mountain, the air is better than the track. And it’s not as hot in summer, too. (Participant 9)

I’m building a house in the mountains and preparing to go to the countryside. I’m going somewhere with clean air, and since it’s the countryside, I can just walk around here and there without any particular plans, you know. And there’s a mountain right behind [our house]. (Participant 20)

Based on the nature of their cancer diagnosis, the participants were sensitive to matters related to the air when exercising. Even in daily life, they paid heed to cleaning and using air purifiers, and meticulously checked fine dust levels before exercising.

I think I’m a bit more sensitive to things related to the air. Anyhow, nobody likes dust, but I think I’ve become more sensitive to things like dust in the house… We had an air purifier before as well, but I think I’m using it more often now, and when I exercise, I worry more about the air now anyway. (Participant 15)

For me, I have a fine dust application. I look at that to check [the dust levels]. I think the [reports] on the internet are generally a little lenient, and so I look at the reports from the WHO, and I always check before I go out to exercise. (Participant 7)

#### Subtheme 2: smart health management and searching for information

The participants were familiar with using digital devices, including smartphones and wearable devices. Therefore, they used these tools to exercise or to measure, set, or complete their activity levels for the day. They checked their exercise levels daily, which encouraged them to exercise regularly and continue the practice.

I had constantly done yoga or gone to the gym before, but I lost a lot of muscle strength when I stopped for a while due to the surgery. I felt like I needed to start exercising again. In fact, this bracelet [wearable device] helped me a lot. When I come regularly and exercise, this lets me see the trends. (Participant 21)

I look everyday [at the smartphone health management app]. When I’m walking and I think, “How many steps have I done?,” I check and it says 8,000 steps, then psychologically I feel like I ought to complete the remaining 2,000 steps. Sometimes I do [exercise], and sometimes I just tell myself, “Let’s do it,” but I end up doing less. On the off chance that I become lazy, this [app] makes me more aware. (Participant 7)

Because this age group was comfortable using the internet, they searched for articles or videos on exercise post-lung cancer, as well as online communities for cancer patients and social networking sites (SNSs). The exercise-related information that participants found allowed them to identify exercise methods that suited them.

Because I heard that I should walk. The professors said that. I heard about patients with cancer while watching a video (on an SNS), and anyway, since it’s my lungs, a lot of people say that I need to do lots of aerobic exercise, whether it’s people around me or on the internet, and I think that’s [one reason] why I did exercise. (Participant 22)

I looked for YouTube videos and there were videos for walking 3 miles at home on days when it was raining or there was too much particulate matter. And so I watched that and I tried it. It was still no fun, but it was better than doing it alone. I usually visit cafes [online communities] rather than searching the internet. I get most of my information from these cafes. (Participant 16)

### Theme 3: conflict between the need to exercise and practical difficulties

The third theme reflected the situation wherein participants perceived the importance of exercise, but they also faced issues in exercising due to various difficulties. Lack of information and guidelines, time restrictions, psychological pressure, and mistaken perceptions collectively created a major challenge to sustaining exercise post-surgery.

#### Subtheme 1: lack of information and guidelines for exercise

The participants needed to accurately identify their post-surgery physical condition and exercise methods before performing resistance or aerobic exercises. However, they could not obtain specific information for this purpose. Many participants could not even distinguish between resistance and aerobic exercise. In addition, community exercise centers or gyms provided the same exercise recommendations for patients with cancer and the general population. Without precise information, some patients even developed issues because they engaged in exercises that were not suitable for their body.

“Just walk for one hour however many times per day” or “Do just this much exercise, and you mustn”t do too much.” If there was something like that it would be quite helpful. But because there aren’t any guides like that, for a long time, I would run diligently, thinking of how I used to be, and I suffered because of it. Patients don’t know. When they take a lung, I don’t know how much it affects me. I don’t know my body, and so I suffered by trying to exercise like I used to. (Participant 2)

It’s important to differentiate between people like us, who’ve had surgery, and the general population. But when you go to a gym, they make you exercise like anyone else. Then it’s often difficult to keep up. And if it’s not fun and you lose interest, you stop going. I think the hospital should teach you about exercise machines or aerobic exercise methods, and help to find the ideal exercise for one’s body. (Participant 8)

Because the participants were not sure what type of exercise was suitable for them, they would experience pain, discomfort, or breathing difficulties during excessive exercise, and not know how to deal with it. When exercise was very distressing, it made participants fear or avoid exercise because they were worried about suffering from the same symptoms again.

At first [they], kept telling me to exercise. Early after surgery, I couldn’t exercise. (Omitted). For the first one to three months, that was my biggest crisis. Mental issues too. When I was out of breath, I really wanted to die. I can’t [exercise] anymore because I’m scared. (Participant 1)

When I go up the mountain, I get a bit out of breath. At first, I would walk and go up a little and I would have breathing difficulties. I would take deep breaths and [say to myself] “That’s right, did you do a bit too much exercise yesterday? Did you go up a bit too high? I think that’s why I’m hurting.” (Participant 19)

#### Subtheme 2: difficulty exercising due to a busy life

Many participants were working in an office or were self-employed, and they continued to work after returning early from surgery. They believed that moving while working was enough, but they also felt uneasy about not exercising.

I do a little bit of yoga, or else I don’t [exercise]. Because I go to the office. I think that’s enough. (Omitted) Sometimes I’m out until almost 12 o’clock at night, and so I can’t exercise. It’s too difficult. (Participant 17)

I’m moving every day because I have to do business. I don’t do any separate exercise. I just walk when it’s close. I feel anxious as well because I can’t exercise. (Participant 12)

#### Subtheme 3: psychological pressure due to the fear of recurrence

The participants experienced a fear of recurrence, thinking that their disease might return if they did not exercise. Because they had early-stage lung cancer and only received surgical treatment, they also thought that continual exercise was everything in terms of post-cancer management. Because they were afraid of recurrence, they felt a sense of pressure that they had to exercise even when they did not want to.

If it was a different part of my body, I would do something else, but because it’s my lungs. I think the air is important, and so I go up the mountain in the morning. Its difficult. Now it’s summer, so it’s hot and I get breathless. But I still go up [the mountain]. If I don’t, I feel anxious for some reason. I think “anxious” is the right expression. If I don’t exercise, I feel anxious. (Participant 4)

It’s been constantly hammered into my head in the ward since the day of the surgery. If I don’t want [the cancer] to come back, I plan to keep [exercising], but that’s difficult. Because it’s not fun. But still, you have to make yourself do it deliberately. (Participant 7)

#### Subtheme 4: mistaken perceptions about exercise

Some participants had misunderstandings about exercise post-surgery, which became a reason for not exercising. Some participants avoided exercise, mentioning problems such as exercise leading to issues for their lungs after surgery, or the possibility of their situation becoming worse due to the environment they exercise in.

First of all, I can’t exercise because I’m scared. I exercised a lot before. I have heard that if you run, it’s not good for your lungs. I heard a lot that being out of breath is not good for you. Now, because I can’t exercise properly, my quality of life has decreased a lot. (Participant 11)

[I’m worried that] if I exercise in winter I’ll get a cough or pneumonia… I saw someone who got pneumonia after surgery. It seemed to be really severe and fatal. After seeing that person, I almost never participate in activities outside. (Participant 14)

## Discussion

The results revealed that middle-aged patients diagnosed with early lung cancer suffer psychological and emotional confusion but recover faster than expected. They view exercise as a priority to prevent recurrence. The findings confirm the need to provide customized exercise information, practical management strategies, and psychological support. They also highlight the importance of developing accurate perceptions regarding exercise.

The participants experienced extreme fear and anxiety about survival and the risk of cancer recurrence immediately after diagnosis. However, with rapid recovery post-surgery, they began to look forward to returning to daily life, and physical recovery led to a positive emotional turnaround. These findings support previous reports that patients with lung cancer experience anxiety immediately after their diagnosis [24]. They are also consistent with studies showing that patients with lung cancer experience fear about disease progression after surgery [25]. However, this study showed that despite the initial psychological crisis, patients’ experience of rapid physical recovery can lead them in a positive direction. In particular, Kong et al. [10] and Marzorati et al. [26] found that when patients who had received surgery for early lung cancer were followed up for a year, their overall health status and physical function improved linearly after surgery [10], and this functional recovery was closely associated with patients’ emotional wellbeing [26]. The participants in the current study also experienced physical recovery and showed a positive emotional shift, as they looked forward to returning to daily life. These results are consistent with previous reports showing that emotional wellbeing has positive effects on long-term recovery and survival for diseases including cancer [27], 28]. The present results also indicate that the post-surgery recovery experiences of middle-aged patients with lung cancer transcend simple physical recovery and act as a catalyst for a psychological transition, which ultimately leads to long-term emotional wellbeing and recovery.

The participants viewed exercise as a priority to prevent disease recurrence and maintain health, and this outlook acted as a core strategy not only driving physical activity but also promoting the formation of a new lifestyle. These results are consistent with previous research focused on long-term cancer survivors, reporting that physical activity levels were significantly correlated with QoL [29]. Interestingly, the participants preferred exercising in natural environments such as the mountains or parks, rather than indoors, due to sensitivity about their pulmonary health. This finding is consistent with that of Lesser et al. [30], who reported that cancer survivors prefer outdoor physical activities and experience psychosocial benefits from these activities. This preference for outdoor exercise has been consistently reported in the general population [31], and among other cancer survivors as well. In one review of 712 cancer survivors, outdoor physical activity (usually walking or Nordic walking) improved physical and mental health [32]. In a study focused on breast cancer survivors, light exercise in a natural environment lowered cholesterol and managed stress than exercise in urban or indoor environments. This reflects the importance of the environment in maximizing the physiological and psychological benefits of exercise [33]. Similarly, the participants in this study preferred outdoor exercise to the point that they sought the countryside, mountains, or places with good air. However, these specific circumstances appeared to stem from lung cancer patients’ sensitivity to air.

As this study was limited to middle-aged adults, the patients were relatively young, actively used tools such as smartphones and wearable devices, and engaged in online communities as part of exercise planning and monitoring. Low [34] reported that in the modern world, digital technologies are useful for monitoring physical activities and supporting self-management in patients with cancer. Allison et al. [35] demonstrated that online peer support improved social support for and psychosocial wellbeing among patients with cancer. The results of the present study suggest that, when designing digital exercise interventions for middle-aged lung cancer survivors, recommending activities in a natural environment and combining wearable devices with online peer support platforms is effective for encouraging exercise continuation and improving QoL.

The participants perceived exercise to be essential for health management and prevention of disease recurrence; however, they faced various difficulties in exercising. This is consistent with a systematic literature review demonstrating that cancer survivors experienced barriers to exercise at multiple levels, with issues including fatigue, lack of time, lack of exercise knowledge, and fear of injury [36]. Blaney et al. [37] demonstrated that these barriers persisted even after completing treatment. Granger et al. [38] reported that even clinicians lacked knowledge about when and how to provide exercise advice to patients with lung cancer, suggesting that there is a scarcity of specialized support systems for these patients. Stout et al. [39] emphasized the need for customized systematic evaluation and exercise recommendations depending on the patients’ functional level, and Bade et al. [40] reported that personalized interventions improved participation rates among patients with advanced lung cancer. The present study highlights the need for multidimensional approaches to designing exercise programs for cancer survivors, combining the provision of personalized information, practical time management strategies, and psychological support, and supporting the development of accurate exercise-related perceptions. For middle-aged patients with lung cancer who are comfortable using digital technologies, exercise interventions and information utilizing these technologies could be an effective approach toward multidimensional support.

This study has some limitations. First, the interviews were conducted in 2017, several years before manuscript submission. Although survivorship care and telehealth-based supportive care have evolved since then, the findings remain relevant because they reflect foundational postoperative exercise experiences, including patients’ perceived benefits, barriers, and support needs, which continue to be reported in more recent oncology literature. Nevertheless, caution is needed when applying these findings directly to current care contexts, particularly those involving digital or telehealth-enabled exercise support. Second, the time since surgery ranged from 1 to 12 months. However, regardless of interview timing, participants commonly recalled their early postoperative period when describing physical discomfort and exercise-related fear, while also reflecting on their current recovery status. This retrospective nature of the accounts allowed shared experiences to emerge across different postoperative stages, and core themes remained consistent across all participants. Future longitudinal studies are warranted to examine how postoperative exercise experiences evolve over time. Third, because the participants were early-stage patients with lung cancer, the study did not capture the characteristics of diverse cancer types and stages. Fourth, because the participants were not sufficiently diverse in terms of occupations or comorbidities, it is difficult to make broad comparisons regarding differences in exercise experiences. Nevertheless, this study provides useful basic data for the development of customized exercise support strategies.

## Conclusions

Among middle-aged patients with early lung cancer, exercise is an important factor not only for physical recovery but also for enabling the recovery of identity and the reconstitution of their lives. These findings highlight the need to understand patients’ experiences beyond symptoms and within their everyday world and meaning structures. By elucidating the exercise-related experiences of patients with early lung cancer, including the need to exercise, specific practical limitations, and a lack of related information, this study offers basic data for the development of customized exercise interventions. In particular, it indicates the need to develop individualized exercise programs that reflect patients’ physical and psychological condition, and to construct reliable, digital systems for the provision of health information. In addition, healthcare organizations and local communities must cooperate to provide patients with lung cancer with access to accurate information about exercise and environments that support sustained adherence to exercise.
